# Autonomous Learning of New Environments with a Robotic Team Employing Hyper-Spectral Remote Sensing, Comprehensive In-Situ Sensing and Machine Learning

**DOI:** 10.3390/s21062240

**Published:** 2021-03-23

**Authors:** David J. Lary, David Schaefer, John Waczak, Adam Aker, Aaron Barbosa, Lakitha O. H. Wijeratne, Shawhin Talebi, Bharana Fernando, John Sadler, Tatiana Lary, Matthew D. Lary

**Affiliations:** Hanson Center for Space Sciences, University of Texas at Dallas, Richardson, TX 75080, USA; captdaveschaefer@gmail.com (D.S.); John.Waczak@utdallas.edu (J.W.); Adam.Aker@utdallas.edu (A.A.); aaronbarbosa.me@gmail.com (A.B.); lhw150030@utdallas.edu (L.O.H.W.); Shawhin.Talebi@utdallas.edu (S.T.); ashen.fernando@utdallas.edu (B.F.); jcs170001@utdallas.edu (J.S.); txl160130@utdallas.edu (T.L.); mdlary@me.com (M.D.L.)

**Keywords:** machine learning, hyper-spectral imaging, robot team, autonomous, UAV, robotic boat

## Abstract

This paper describes and demonstrates an autonomous robotic team that can rapidly learn the characteristics of environments that it has never seen before. The flexible paradigm is easily scalable to multi-robot, multi-sensor autonomous teams, and it is relevant to satellite calibration/validation and the creation of new remote sensing data products. A case study is described for the rapid characterisation of the aquatic environment, over a period of just a few minutes we acquired thousands of training data points. This training data allowed for our machine learning algorithms to rapidly learn by example and provide wide area maps of the composition of the environment. Along side these larger autonomous robots two smaller robots that can be deployed by a single individual were also deployed (a walking robot and a robotic hover-board), observing significant small scale spatial variability.

## 1. Introduction

This paper describes a robotic team that can rapidly learn new environments. The system that is described here demonstrates a flexible paradigm that is easily scalable to multi-robot, multi-sensor autonomous teams.

The inspiration for this autonomous robotic is the automation of what is currently done manually in the production of remote sensing satellite data products. The typical timescale from starting work on a new remote sensing data product to its operational readiness is at least a couple of years, but, more typically, a decade or more. A key part of this substantial time delay is due to the time that is taken for the collection of the relevant training data. Hence, our goal was to reduce this timescale to be near real time by utilising an autonomous robotic team that can both collect the training data, and then in real time process and stream the remote sensing data products.

A case study is described in detail for the rapid characterisation of the aquatic environment. Other authors have described, in detail, various configurations of autonomous robots, for example [1,2,3,4,5,6]. Here, we leverage our past experience over the last two decades in pioneering the use of machine learning for providing and calibrating remote sensing data products [7,8,9,10,11,12,13,14,15,16,17,18,19,20,21,22,23,24] and use it to inform the design and operation of the robotic team.

The aquatic environment was chosen, as it includes extra challenges with regards the ease of access, further demonstrating the value of the approach. When considering the usefulness of being able to conduct such rapid surveys, it is worth noting that, for just the oil spill response use case alone, the National Academy of Sciences estimates that the annual oil spill quantities range from 1.7 million tons to 8.8 million tons. Over 70% of this release is due to human activities. The result of these spills include dead wildlife, contaminated water, and oil-covered marshlands [25,26,27,28]. Accordingly, being able to rapidly survey such areas to guide clean-up operations is of considerable use. It is also of use in a wide variety of contexts, from general environmental surveys, to studying harmful algal blooms, to the clean-up operations after natural disasters, such as hurricanes, etc.

**Figure 1:**
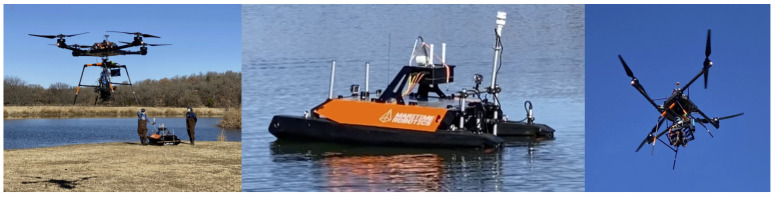
Photographs of the robot team during a Fall 2020 deployment in North Texas.

In the example that is described in this paper, the fully autonomous team includes a robotic boat that carries a suite of sensors to measure water composition in real time as well as a sonar, and an autonomous UAV equipped with a down-welling irradiance spectrometer, hyper-spectral, and thermal imagers, together with an onboard Machine Learning (ML) capability. Figure 1 shows photographs of the robot team during a December 2020 deployment in North Texas.

Besides this capability being useful by itself, there is a wider significance for earth observing satellite missions. A key component to each and every space agency earth observation mission is the delivery of a suite of data products and the calibration/validation of these products. The demonstrated paradigm can reduce the time and cost of producing new remote sensing data products, while increasing the functionality and data quality and providing new real-time automated calibration/validation capabilities.

The approach also provides enhanced capabilities for real-time onboard data product creation, reducing product delivery latency. The end-to-end demonstration uses all off-the-shelf components, representing a reduction in costs and risk when prototyping new mission concepts.The use of embedded machine learning is a key element, so we will refer to the approach as Rapid Embedded Prototyping for Advanced Applications (REPAA).

### Hyper-Spectral Imaging

**Figure 2:**
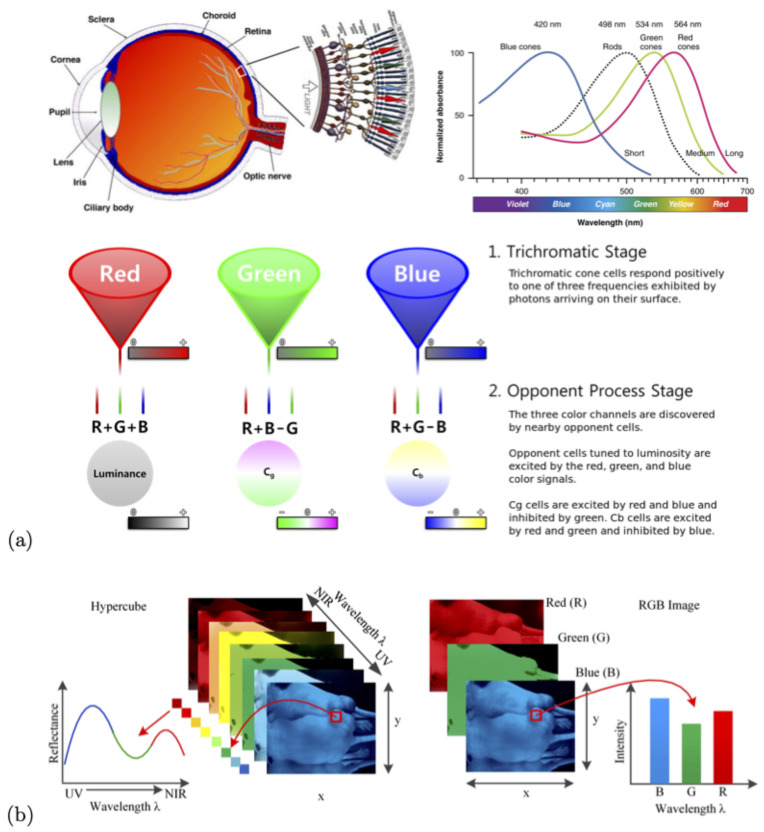
Panel (**a**) Trichromatic cone cells in the eye respond to one of three wavelength ranges (RGB). Panel (**b**) shows a comparison between a hyper-spectral data-cube and RGB images.

The human eye perceives the color of visible light in three bands using the cones, the photoreceptor cells in the retina (Figure 2). These three broad bands are red (centered on 564 nm), green (centered on 534 nm), and blue (centered on 420 nm). By contrast, instead of using just three broad bands, hyper-spectral cameras divide the spectrum into a very large number of narrow bands, in our case 463 bands from 391–1011 nm. A hyper-cube is a three-dimensional dataset that consists of a stack of two-dimensional image layers each for a different wavelength. Hence, for each pixel in the image, we have a multi-wavelength spectra (spectral signature). This is schematically shown in the lower left of Figure 2. On the right, we see a conventional RGB color image with only three bands, images for red, green, and blue wavelengths.

**Figure 3:**
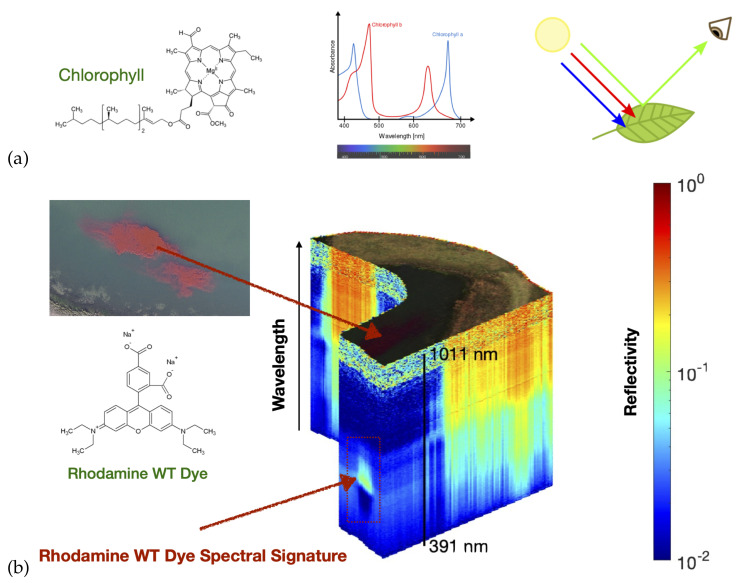
Panel (**a**) Chemicals absorb light in a characteristic way. Their absorption spectra is a function of their chemical structure. For every pixel we measure an entire spectrum with a hyper-spectral camera so we can identify chemicals within the scene. Panel (**b**) shows an example hyper-spectral data cube collected in North Texas on 23 November 2020. This particular data cube includes a simulant release, Rhodamine WT. The top layer of the hyper-spectral data cube shows the regular RGB image, the 462 stacked layers below show the reflectivity (on a log-scale) for each wavelength band between 391 and 1011 nm.

Chemicals absorb light in a characteristic way. Their absorption spectra is a function of their chemical structure. Figure 3a shows the structure of chlorophyll and the associated absorption spectra. So that we can accurately calculate the reflectivity at each wavelength our autonomous UAV measures both the incident downwelling irradiance of incident solar radiation and a hyper-spectral imager pointed directly down at the earth’s surface below the UAV. For every pixel we measure an entire spectrum with a hyper-spectral camera so we can identify chemicals within the scene.

Figure 3b shows an example reflectivity hyper-spectral data cube collected during a robot team deployment in North Texas during November 2020. This data cube includes the area where an inert dye was released to test the system. The dye used was Rhodamine WT, a fluorescent, xanthene dye, which has long been used as a hydrologic tracer in surface water systems. The spectral signature of the dye is clearly visible in the hyper-spectral data cube. The top layer of the hyper-spectral data cube shows the regular RGB image, the 463 stacked layers below show the reflectivity (on a log-scale) for each wavelength band between 391 and 1011 nm.

## 2. Materials and Methods

All of the data for the machine learning data product creation were collected in a coordinated automated manner using the autonomous robotic team.

The most time consuming process in building this robotic team was finding the appropriate off the shelf components to implement the prototype. So here we have provided the full detailed recipe on the autonomous robotic team in the hope that it will facilitate the research of others.

An overview of the robotic team members and their sensor payloads is as follows.

### 2.1. Robotic Vehicles

A Maritime Robotics Otter (https://www.maritimerobotics.com/otter, accessed 5 January 2021) autonomous boat was used. With a footprint of only 200 × 108 × 81.5 cm, a weight of 55 kg, and dual electrical fixed thrusters, it is an easily deployable asset that can be transported in a van or even within normal airliners to a survey site. With a cruise speed of two knots, it has a duration of 20 h from one charge of the batteries. It can use WiFi, cellular, and an optional AIS receiver for communication to the control station.

A Freefly Alta-X (https://freeflysystems.com/alta-x, accessed 5 January 2021) autonomous professional quad-copter was used. It was specifically designed to carry cameras, with a payload capacity of up to 35 lb, a long range data link, and autonomy provided by the Open PX4 flight stack. The open source QGroundControl software was used to control the autonomous operations (https://freeflysystems.com/support/alta-pro-support, accessed 5 January 2021). QGroundControl is available for Mac, Windows, iOS, and Android.

All of the robotic team members carry a high-accuracy GPS and INS, so that every data point can be geo-located and time stamped. Each of the robots can also join the same network which connects the robots and their ground-control stations. Our robots use long-range Ubiquiti 5 GHz LiteBeam airMAX WiFi (https://www.ui.com, accessed 5 January 2021). The airMAX Time Division Multiple Access (TDMA) protocol allows for each client to send and receive data using pre-designated time slots that are managed by an intelligent AP controller. This time slot method eliminates hidden node collisions and maximizes airtime efficiency. This WiFi network is connected to the internet using a Cradlepoint cellular modem (https://cradlepoint.com, accessed 5 January 2021).

This network also includes a local Synology network-attached storage (NAS) (https://www.synology.com, accessed 5 January 2021) device in the robot team control trailer, which, in real-time, syncs the data that were collected to the NAS in our home laboratory in the university.

### 2.2. Boat Sensors

The robotic boat payload included a BioSonics MX Aquatic Habitat Echosounder sonar for rapid assessment and mapping of aquatic vegetation, substrate and bathymetry (https://www.biosonicsinc.com/products/mx-aquatic-habitat-echosounder/, accessed 5 January 2021). Three Eureka Manta-40 multi-probes (https://www.waterprobes.com/multiprobes-and-sondes-for-monitori, accessed 5 January 2021), a Sequoia Scientific LISST-ABS acoustic backscatter sediment sensor (https://www.sequoiasci.com/product/lisst-abs/, accessed 5 January 2021), and an Airmar Technology Corporation 220 WX ultra-sonic weather monitoring sensor (https://www.airmar.com/weather-description.html?id=153, accessed 5 January 2021).

The first Manta-40 multi-probe included sensors for temperature and turbidity and Turner Designs Cyclops-7 submersible Titanium body fluorometers (https://www.turnerdesigns.com/cyclops-7f-submersible-fluorometer, accessed 5 January 2021) for Chlorophyll A, Chlorophyll A with Red Excitation, Blue-Green Algae for fresh water (Phycocyanin), Blue-Green Algae for salt water (Phycoerythrin), and CDOM/FDOM. The second Manta-40 multi-probe included sensors for temperature, conductivity (with specific conductance, salinity, and total dissolved solids, TDS), pH (with separate reference electrode), optical dissolved-oxygen, turbidity, and Ion Selective Electrodes by Analytical Sensors and Instruments (http://www.asi-sensors.com/, accessed 5 January 2021) for ammonium (NH${}_{4}^{+}$), bromide (Br${}^{-}$), calcium (Ca${}^{++}$), chloride (Cl${}^{-}$), nitrate (NO${}_{3}^{-}$), and sodium (Na${}^{+}$). The third Manta-40 multi-probe included sensors for temperature, turbidity, a total dissolved gas sensor, and Turner Designs Cyclops-7 submersible Titanium body fluorometers for optical brighteners, crude oil, refined fuels, and tryptophan.

In addition, a portable Membrane Inlet Mass Spectrometer (MIMS) designed and built by Prof. Verbeck of the University of North Texas is available (but not used in these deployments) to switch every 3 s between sampling the water composition and the air composition.

### 2.3. Aerial Sensors

The aerial vehicle used a Gremsy H16 gimbal (https://gremsy.com/gremsy-h16, accessed 5 January 2021) that was made with aircraft grade aluminum and carbon fiber to carry a Resonon Visible+Near-Infrared (VNIR) Pika XC2 (https://resonon.com/Pika-XC2, accessed 5 January 2021) hyper-spectral camera (391–1011 nm) with a Schneider Xenoplan 1.4/17 mm lens, and a FLIR Duo Pro R, (640 × 512, 25 mm, 30 Hz) combining a high resolution, radiometric thermal imager, 4K color camera, and a full suite of onboard sensors (https://www.flir.com/products/duo-pro-r/, accessed 5 January 2021). On the top of the quad copter there is a sky facing Ocean Optics UV-Vis-NIR spectrometers measuring the incident down-welling irradiance, allowing us to calculate reflectance.

### 2.4. Geo-Rectification

The hyper-spectral data cubes collected are very large and written in real time to the solid-state disk (SSD) that was attached to the Resonon Pika XC2. The Camera SSD is exported as a Network File System (NFS) mount, so that a second onboard computer can geo-rectify the hyper-spectral data cubes as they are created, in order to facilitate the real-time processing of these files. These hyper-spectral data cubes provide a visible and near infrared spectrum (391–1011 nm) for each pixel. Once these data cubes are geo-rectified in real-time, they are available for onboard machine learning using edge computing onboard the aerial vehicle.

### 2.5. Machine Learning

The accurate geo-tagging and time stamping of all data from all members of the robot team allows for the automation of the machine learning data product creation. For every location at which the robotic boat sampled the in-situ water composition, we associate a VNIR remotely sensed spectrum (391–1011 nm) that is provided by the hyper-spectral data cubes collected by the aerial-vehicle. This data are then be used for multi-variate non-linear non-parametric machine learning, where the inputs are the spectrum, in this case 462 values from the 391–1011 nm spectra, and the outputs are each of the values measured in-situ by the robotic boat. A variety of machine learning approaches were used. These approaches included shallow neural networks with hyper-parameter optimization, ensembles of hyper-parameter optimized decision trees, gaussian process regression with hyper-parameter optimization, and a super-learner, including all of the previously mentioned approaches. Each empirical non-linear non-parametric fit is evaluated by constructing both a scatter diagram and a quantile-quantile plot of the values estimated by the machine learning model plotted against the actual values in the independent validation dataset.

The use of machine learning in this study builds on our heritage of using machine learning for sensing applications over the last two decades [7,8,9,10,11,12,13,14,15,16,17,18,19,20,21,22,23,24].

## 3. Learning Modes

**Figure 4:**
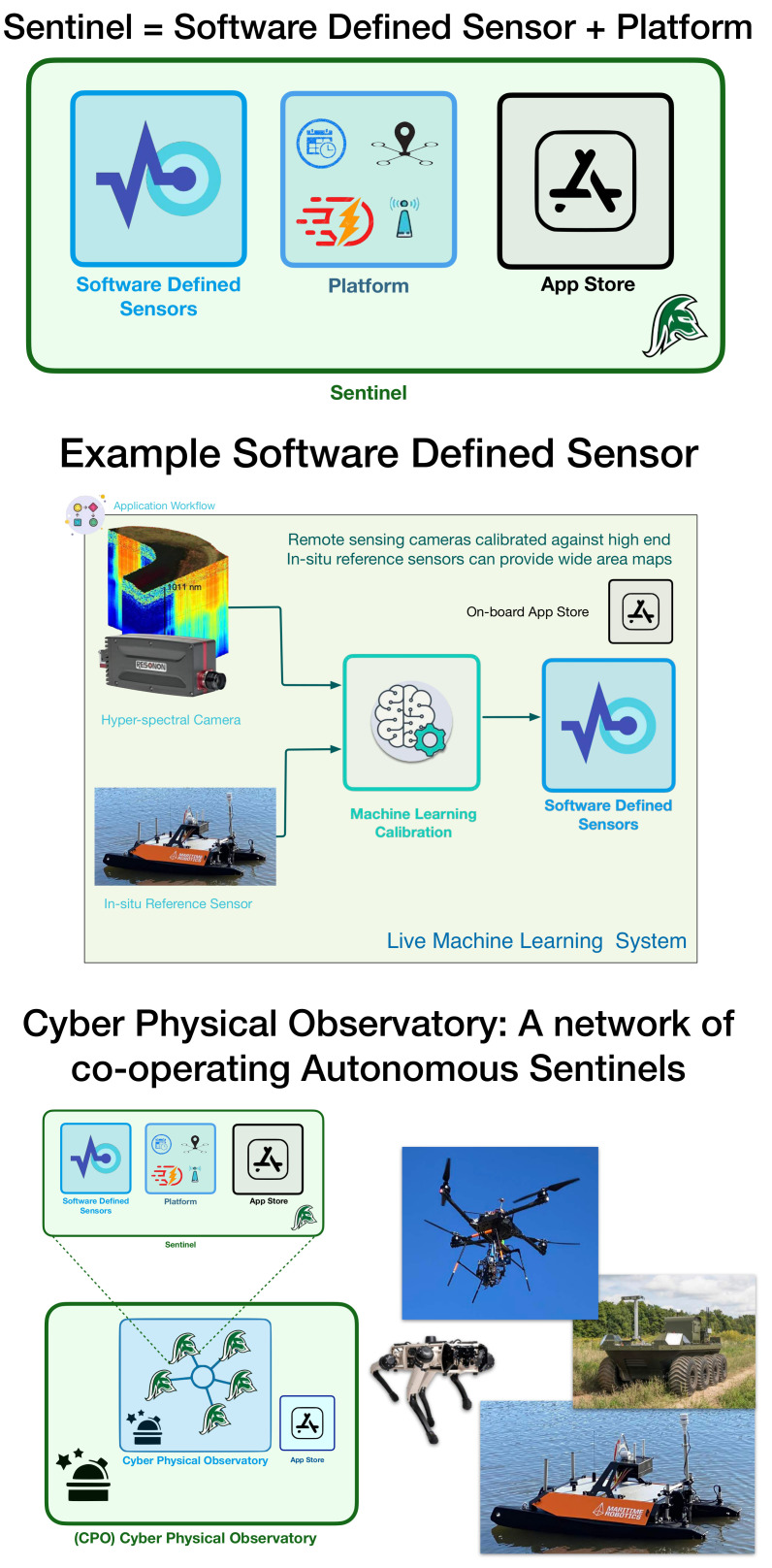
The Cyber Physical Observatory is a collection of sentinels that provide real-time data. A Sentinel is a Software Defined Sensor that is mounted on a Platform. A Platform supplies the Software Defined Sensor with power, timestamps for all observations, communication, and mobility where applicable. A Software Defined Sensor is a smart sensor package that combines a physical sensing system with machine learning providing a variety of calibrated data products that can be updated via an app store.

We designed each component of our system to be flexible for different scenarios and deployment configurations. The entire system is called a Cyber Physical Observatory (Figure 4). A few basic definitions/descriptions are helpful in appreciating the benefits of this. The Cyber Physical Observatory is a collection of sentinels and/or robot teams that provide real-time data and actionable insights, and whose capabilities can be updated via an app store. The Robot Team is a collection of co-operative autonomous sentinels. A Sentinel is a Software Defined Sensor that is mounted on a Platform. A Platform supplies the Software Defined Sensor with power, timestamps for all observations, communication, and mobility where applicable. In some of our other applications, these even include wearable sensors. A Software Defined Sensor is a smart sensor package that combines a physical sensing system with software/machine learning, providing a variety of calibrated data products that can be updated via an app store.

**Figure 5:**
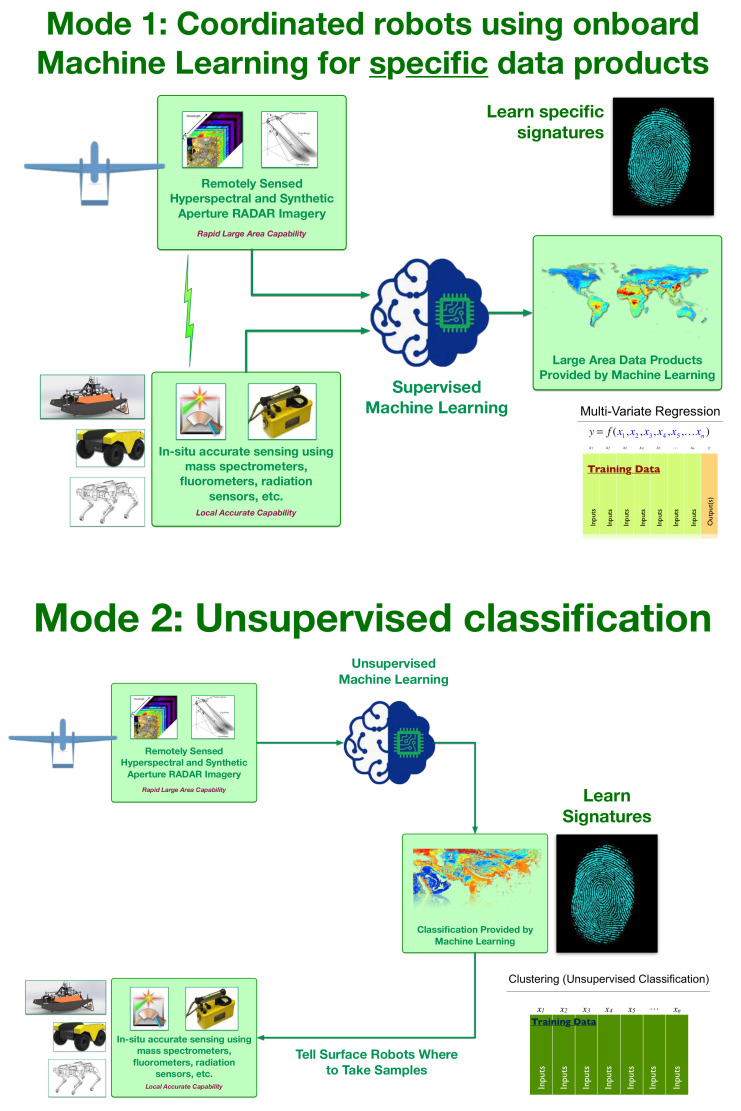
The autonomous robotic team operates in two modes. **Mode 1**: Coordinated robots using onboard Machine Learning for specific data products. **Mode 2**: Unsupervised classification.

Two distinct machine learning modalities are useful when trying to rapidly learn new environments (Figure 5). Mode 1: Coordinated robots using onboard Machine Learning for specific data products. Mode 2: Unsupervised classification.

**Figure 6:**
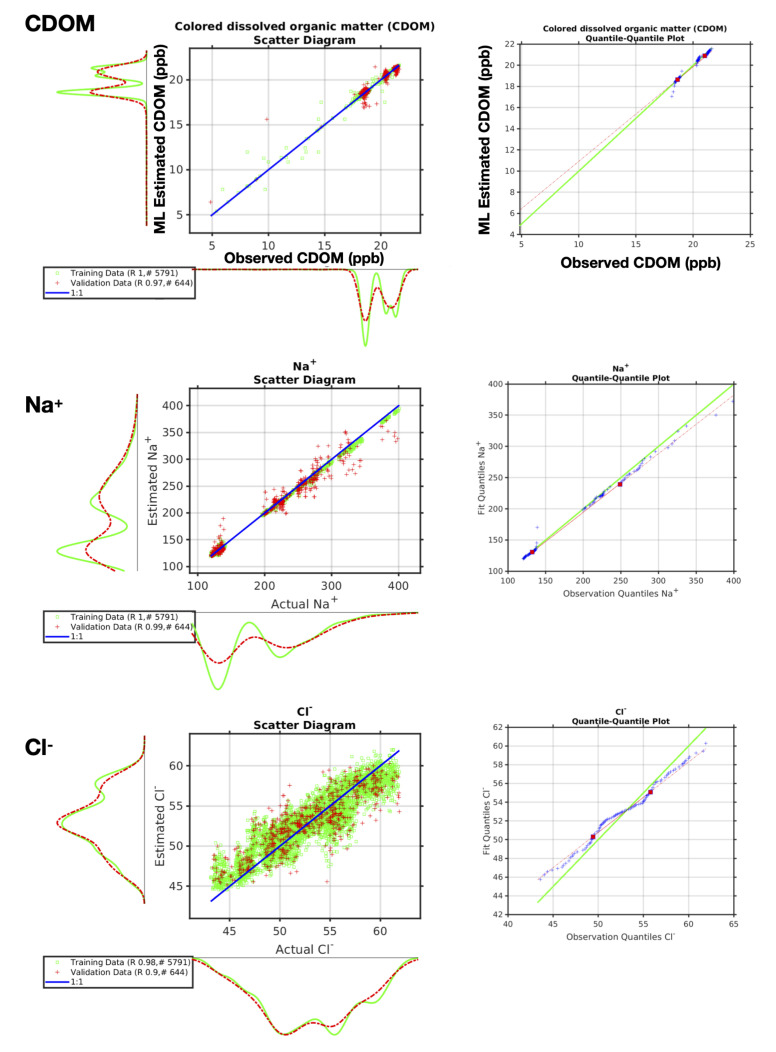
Machine learning performance quantified by both scatter diagrams and quantile-quantile plots utilising data collected autonomously by the robot team during three exercises during November and December 2020 in North Texas. The three examples shown here are for CDOM, Na^+^, and Cl^−^. The scatter diagrams show the actual observations (mg/L) on the *x*-axis and the machine learning estimate on the *y*-axis. The green curves are for the training data, the red for the independent validation. The legend shows the number of points in the training and validation datasets and their associated correlation coefficients. The quantile-quantile plots show the observation quantiles on the *x*-axis and the machine learning estimate quantiles on the *y*-axis.

In Mode 1, the robot team members rapidly collect the machine learning training data in a carefully coordinated manner. For our example deployment in North Texas during the Fall of 2020, over a period of about fifteen minutes, thousands of precisely collocated measurements were made by the robotic team. The robotic boat autonomously measures in-situ ground truth of a large array of parameters using the sensors described above, while the robotic aerial vehicle gathered remotely sensed observations of exactly the same locations using hyper-spectral and thermal imaging. These remotely sensed observations could be readily extended to cover a wider wavelength range and include Synthetic Aperture Radar (SAR). Once the training data are acquired, the machine learning algorithms can rapidly learn the mapping from the remotely sensed observations to the in-situ ground truth. Figure 6 shows three different examples of the validation of these autonomously acquired machine learning data products being independently verified while using scatter diagrams and quantile-quantile plots.

**Figure 7:**
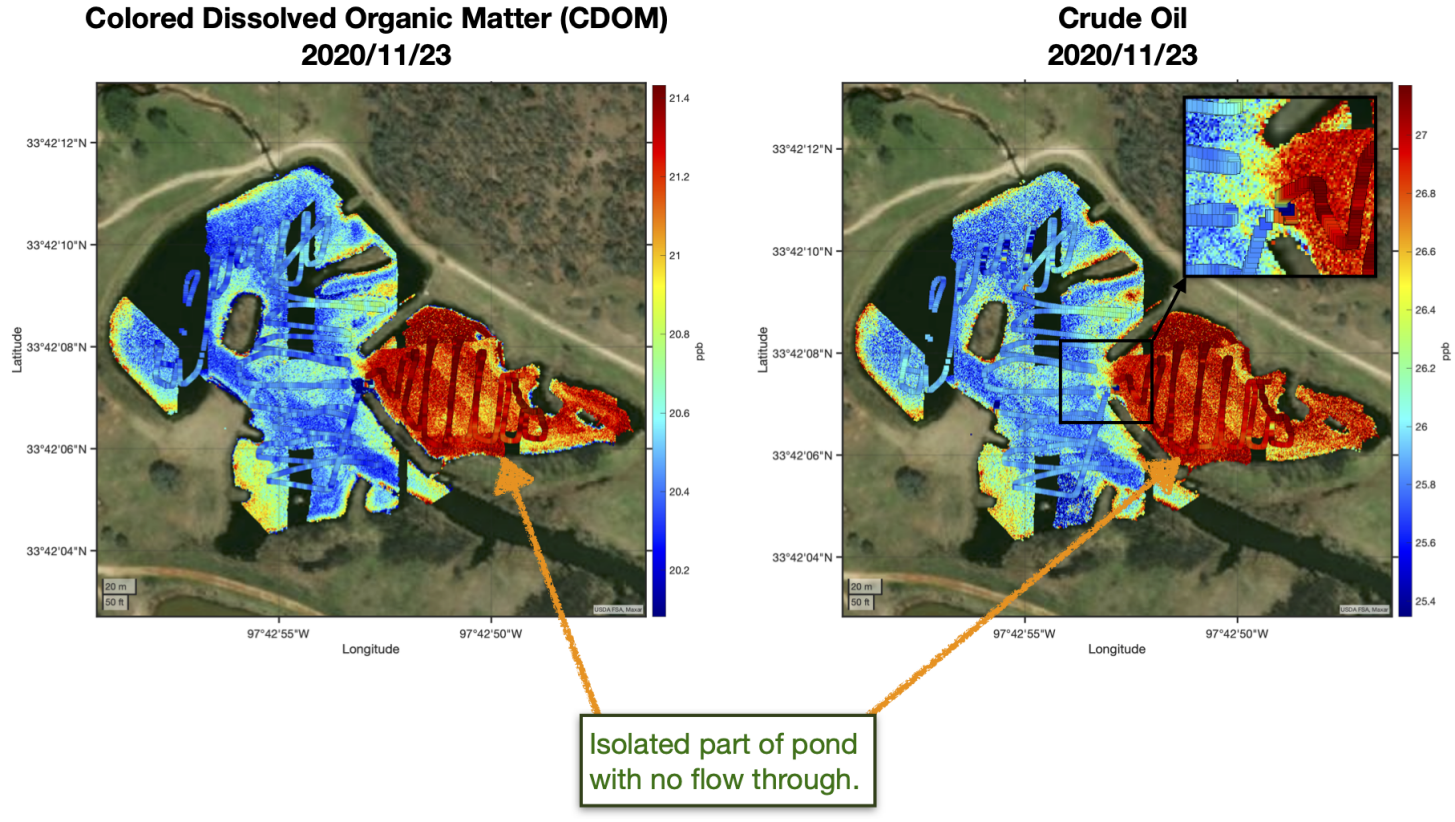
Example crude oil and colored dissolved organic mater (CDOM) data collected autonomously by the robot team on November 23, 2020 in North Texas. The maps show the CDOM and crude oil estimated from the hyper-spectral imager using machine learning as the background colors and the actual in-situ boat observations as the overlaid color filled squares. Note that the isolated part of the pond, which has now fresh water in-flux, has higher levels of CDOM and crude oil with a sharp gradient across the inlet in both the estimates using the hyper-spectral image and the boat observations.

Once the machine learning algorithm(s) have been trained, they can then be used to rapidly provide wide-area maps with just the remotely sensed observations. Two examples of this are shown in Figure 7. These can be processed onboard the aerial vehicle and the results streamed in real-time to the ground control station. The robotic boat can then be autonomously tasked to verify the wide area maps by collecting independent validation data.

In Mode 2, we would like to perform a fine-grained multi-class surface classification of the entire domain. This is done by providing the remotely sensed data (in this case, the hyper-spectral and thermal imagery) to an unsupervised classification. The unsupervised machine learning characterises the distinct regions and zones in the area of interest. This can be particularly useful when trying to identify the location of particular contaminants, suggesting the optimum sampling patterns that are required beyond the usual clover leaf, star, or box patterns used for contaminant searches.

## 4. Results

Over a period of just a few minutes, we acquire thousands of training data points. This training data allows for our machine learning algorithms to rapidly learn by example. The machine learning fit used here is an optimized ensemble of regression trees [29,30,31] with hyper-parameter optimization [32] implemented in Matlab version 2021a (https://www.mathworks.com, accessed 5 January 2021) using the function fitrensemble with all hyper-paramter optimization selcted and parallel processing enabled. A loop is executed over all the variables that were measured by the robotic that we would like to estimate using the hyper-spectral imagery.

A balanced training dataset is constructed for each of these variables. This is done by considering each input and output variable in the training dataset in turn and calculating *n* percentiles, from each of these *n* percentile ranges covering the entire PDF, from each percentile range we select *m* random values (where m<n) for the training and a different set of random values for independent validation.

Figure 6 shows an example of the colored dissolved organic mater (CDOM) data collected autonomously by the robot team on 23 November 2020 in North Texas, along with some of the aqueous ion data. The panel shows a scatter diagram of the actual observations on the *x*-axis and the machine learning estimate on the *y*-axis. The green curves are for the training data, the red for the independent validation. On each axis, we also show the associated PDFs. The ideal result is shown in blue (a slope of 1 and an intercept of zero for the scatter diagram).

Figure 7 shows maps of the CDOM and crude oil concentration estimated while using the machine learning as the background colors and the actual in-situ boat observations as the overlaid color filled squares. Note that the isolated part of the pond that now has fresh water in-flux has higher levels of CDOM and crude oil with a sharp gradient across the inlet in both of the estimates using the hyper-spectral image and the boat observations. We note that there is good agreement between the machine learning estimate and the actual in-situ boat observations.

## 5. Discussion

### 5.1. Limitations

The fidelity of the data products that are provided by the autonomous robotic team is limited by the training data that it is able to acquire. For example, our remote sensing hyper-spectral camera in the demonstration use case presented here observes the spectral region 391–1011 nm. It would be useful to extend this spectral region, so that we can see more chromophores, and to extend the type of remote sensing imaging, e.g., to include Synthetic Aperture RADAR (SAR).

It would also be useful for the boat to have larger pontoons, so that it can carry our mass-spectrometer that can sample both the air and water, switching between the two inlets every three seconds.

We would also like to extend the machine learning approaches to include Physics Based machine learning, such that the machine learning is constrained by known physical principles.

### 5.2. Automating Data Product Creation

A key factor in providing remotely sensed water composition products is providing a comprehensive database of water composition (e.g., SeaBASS, the publicly shared archive of in-situ oceanographic and atmospheric data maintained by the NASA Ocean Biology Processing Group https://seabass.gsfc.nasa.gov, accessed 5 January 2021). The cost of making the measurements of ocean composition can be substantial, because it involves a significant ship time as well as a large support team. Secondly, because the satellites are in a fixed orbit with a fixed viewing geometry, the number of coincidences between the shipboard water observations and the orbiting satellite observations are, by definition, limited. Typically several thousand coincident observations are used in the tuning and creation of a NASA ocean data product. In the REPAA approach, the entire system can be automated and objectively optimized. Thus, with a data rate of one observation every second, in a matter of hours we can gather tens of thousands of observations in a totally automated, fully coordinated manner, as was demonstrated in North Texas during November and December 2020 (Figure 1). There is explicit coordination between the water observations that were taken from the robotic boat and the continuous aerial observations made by the robotic aerial vehicle carrying a hyper-spectral imager. The system can be deployed to very diverse environments across a matter of just weeks to months, so, over a matter of just weeks to months, millions of coordinated, precisely coincident records can be made. Furthermore, we have previously demonstrated, the data can be randomly partitioned into training and independent validation sets, and using the onboard machine learning, transformed into optimal water composition data products, using many orders of magnitude more observations than before at a fraction of the cost and in a fraction of the time.

Aurin et al. [33] provides one of the most comprehensive training datasets to date for Chromophoric Dissolved Organic Matter (CDOM). Their Global Ocean Carbon Algorithm Database (GOCAD) for Chromophoric Dissolved Organic Matter (CDOM) encompasses 20,000–100,000+ records (depending on the variable considered) and it is based on oceanographic campaigns that were conducted across the world over the past 30 years at great expense. In contrast, the autonomous robotic team can collect around 20,000+ precisely coordinated training records per hour. By design, the robotic team makes precisely coordinated overpasses of exactly the same locations; this leads to providing a training dataset with a high data rate. By deploying the team on multiple occasions at a diversity of locations, one can rapidly build a comprehensive training dataset.

**Figure 8:**
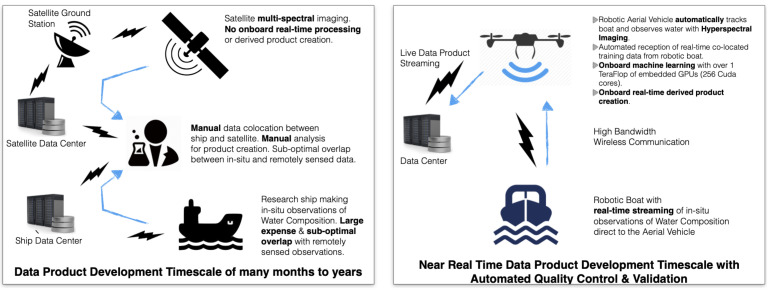
Schematics illustrating the traditional approach to creating remote sensing data products (**left**) and that used in this study (**right**).

The traditional approach for creating remote sensing data products, as shown on the left of Figure 8, is compared with the approach that was used in this study, as shown on the right. Using the REPAA approach, data collection and the creation of derivative data products can be carried out on the same day, for example, in the December 2020 exercises in North Texas (Figure 1).

### 5.3. Improving Product Quality & Automating Cal/Val

Critical in improving product quality is the comprehensive training data set, which spans as much parameter space and variability that is actually found in the real world. This necessitates making observations in a large number of diverse contexts. Being able to make these observations with such a highly automated platform is a tremendous step forward and it costs less. In summary, our robotic platform can address the issue of small scale variability encountered across a satellite pixel. These capabilities assist in continuing validation/quality control and it can help to optimize the waveband selection for future satellite instruments and missions.

### 5.4. Reducing Latency for Product Delivery as Well as Mission Risk, Cost, Weight and Size

Utilizing new embedded onboard processing (1 TeraFlop weighing just 88 g with a size of only 87 mm × 50 mm) for real-time onboard processing leads to reducing the latency in product delivery from hours/days to just the downlink time. The product delivery latency can be critical for decision support applications, such as oil spills, or other disaster response applications, and for routine forecasting and data assimilation applications. A risk reduction is also realized, by the ability to first deploy an end-to-end demonstrator, while using entirely commercial off the shelf components and low cost aerial vehicles, with all software being made Open Source.

### 5.5. Onboard App Store

There is currently a rapid enhancement in both observing capabilities and the embedded computing power from miniaturized low power devices. As these enhanced observing capabilities become routinely available on small cubesats (like hyperspectral imaging), the number of possible uses and applications for societal benefit grows. However, so does the bandwidth that is required for the downlink of the hyperspectral datacubes. Hence, the possibility of onboard processing, for example, using embedded machine learning, means that product creation can occur directly onboard the cubesats and then streamed live via the downlink. This reduces the latency of product creation and the bandwidth that is needed for the downlink. The next logical step, then, of a rapid prototyping and agile workflow, is an onboard app store, where new data products can be deployed to the remote sensing platform for seamless use onboard. A formalized development, testing, and deployment workflow with an app store facilitates an Earth-observing system that responds to the rapidly changing societal needs while maintaining a rigorous approach to validation. This onboard app store can leverage the smart automated code generation that already exists off the shelf and is now routinely used for automobiles and aircraft across the world. The time has also come for this to be the standard paradigm for earth observation.

### 5.6. Smaller Robots

**Figure 9:**
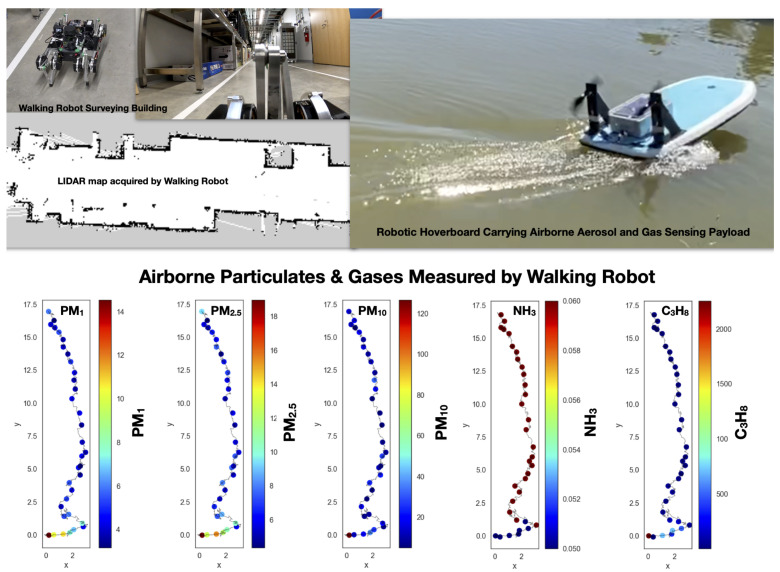
Photographs of the smaller walking robot (from Ghost Robotics) and a robotic hover-board (conceived and built by Aaron Barbosa). For illustrative purposes both of these small robots carried exactly the same payload of sensors measuring the size spectrum of airborne particulates in the size range 0.3–43 microns and the abundance of a selection of gases. The laser scanner onboad the walking robot acquired a map of the vicinity, while also measuring in-situ the atmospheric composition, finding very localized changes in the abundance of the airborne particulates of various sizes.

There is also value in smaller robots that are easy to transport by a single individual. Figure 9 shows photographs of the smaller walking robot (from Ghost Robotics) and a robotic hover-board (conceived and built by Aaron Barbosa) that we deployed along size the larger autonomous robotic team for illustrative purposes. The walking robot and robotic hover-board both carried exactly the same payload of sensors that could be rapidly switched between the robots. The sensing payload measured, every few seconds, the full size spectrum of airborne particulates in the size range 0.3–43 microns and the abundance of a selection of gases. The laser scanner onboad the walking robot acquired a map of the vicinity, while also measuring in-situ the atmospheric composition, finding very localized changes in the abundance of the airborne particulates of various sizes.

## 6. Conclusions

This paper described and demonstrated an autonomous robotic team that can rapidly learn the characteristics of environments that it has never seen before. The flexible paradigm is easily scalable to multi-robot, multi-sensor autonomous teams, and it is relevant to satellite calibration/validation and the creation of new remote sensing data products. A case study was described for the rapid characterisation of the aquatic environment; over a period of just a few minutes, we acquired thousands of training data points. This training data allowed our machine learning algorithms to rapidly learn by example and provide wide area maps of the composition of the environment. Alongside these larger autonomous robots, two smaller robots that can be deployed by a single individual were also deployed, a walking robot and a robotic hover-board, each measuring the full size spectrum of airborne particulates in the size range of 0.3–43 microns and a selection of gases. Significant small scale spatial variability was evident in these hyper-localized observations.

## Data Availability

Not applicable.

## Abbreviations

The following abbreviations are used in this manuscript:

CDOM	Chromophoric Dissolved Organic Matter
GOCAD	Global Ocean Carbon Algorithm Database
GPS	Global Positioning System
INS	Inertial Navigation System
MIMS	Membrane Inlet Mass Spectrometer
ML	Machine Learning
NASA	The National Aeronautics and Space Administration
NFS	Network File System
REPAA	Rapid Embedded Prototyping for Advanced Applications
SeaBASS	SeaWiFS Bio-optical Archive and Storage System
SSD	Solid State Disk
UAV	Unmanned Aerial Vehicle
VNIR	Visible and Near-Infrared
